# The evaluation of protective effect of lycopene against genotoxic influence of X-irradiation in human blood lymphocytes

**DOI:** 10.1007/s00411-017-0713-6

**Published:** 2017-09-14

**Authors:** Aneta Gajowik, Małgorzata M. Dobrzyńska

**Affiliations:** Department of Radiation Protection and Radiobiology, National Institute of Public Health-National Institute of Hygiene, 24 Chocimska Street, 00-791 Warsaw, Poland

**Keywords:** Lycopene, Ionizing radiation, Damage DNA, Antioxidant

## Abstract

Many studies suggest that exogenous antioxidants may protect cells against DNA damage caused with ionizing radiation. One of the most powerful antioxidants is lycopene (LYC), a carotenoid derived from tomatoes. The aim of this study was to investigate, using the comet assay, whether LYC can act as protectors/modifiers and prevent DNA damage induced in human blood lymphocytes, as well as to mitigate the effects of radiation exposure. In this project, LYC, dissolved in DMSO at a concentration of 10, 20 or 40 μM/ml of cell suspension, was added to the isolated lymphocytes from human blood at appropriate intervals before or after the X-irradiation at doses of 0.5, 1 and 2 Gy. Cell viability in all groups was maintained at above 70%. The results showed the decrease of DNA damage in cells treated with various concentrations of LYC directly and 1 h before exposure to X-rays compared to the control group exposed to irradiation alone. Contrary results were observed in cells exposed to LYC immediately after exposure to ionizing radiation. The studies confirmed the protective effect of LYC against DNA damage induced by ionizing radiation, but after irradiation the carotenoid did not stimulate of DNA repair and cannot act as modifier. However, supplementation with LYC, especially at lower doses, may be useful in protection from radiation-induced oxidative damage.

## Introduction

Ionizing radiation (IR) has always accompanied humans. It has been present in the life of our planet since the beginning of its existence. In the twentieth century, man also introduced a number of sources of ionizing radiation like X-ray sources, accelerators, artificial radionuclides and nuclear reactors. Nuclear atmospheric tests, nuclear accidents and nuclear energy production can be sources of low and high doses of ionizing radiation that can cause damage to the human DNA (Pattison et al. 1996; Pattison 1999).

Ionizing radiation produces free radicals, such as reactive oxygen species (ROS), containing unpaired electrons or singlet oxygen, which tend to be highly chemically reactive (Burnham 2001). ROS can react with lipids, proteins and nucleic acids (Fabre et al. 2011; Hall and Giaccia 2006; Riley 1994). These reactions cause oxidative stress and damage, in particular a variety of DNA lesions, like oxidized DNA bases, abasic sites, single-strand breaks and double-strand breaks (Croteau and Bohr 1997; Lu et al. 2010). IR may generate primary reactive free radicals with an extremely short than half-life and a distance of penetration in the range of a micrometres. These factors cannot reach non-irradiated cells. However, electronic spin resonance experiments have shown that long-lived radicals with a half-life ~20 h are also produced in irradiated cells (Koyama et al. 1998). These long-lived radicals can travel in the body and induce DNA damage in non-irradiated cells. Given that their oxidizing power is not as high as that of primary radicals, DNA damage induced by the secondary radicals may not be sufficient to stop DNA replication allowing high-fidelity DNA repair. Hence, they can lead to the amplification of the altered DNA in successive cell generations, and finally to mutations and cell transformation (Azzam et al. 2003; Kumagai et al. 2003; Lala and Chakraborty 2001).

It has been demonstrated that oxidative stress causes long-lasting changes after radiation exposure, possibly due to further generation of ROS and nitrogen radicals (RNS). Interestingly, these modifications occur both in the exposed cells and in their progeny. Oxidative stress may also spread from targeted cells to non targeted bystander cells by means of intracellular control mechanisms (Mitchell et al. 2004; Sawant et al. 2001). DNA damage caused by oxidative stress may lead to mutations that inactivate tumour suppressor genes and activate oncogenes (Dreher and Junod 1996; Jungst et al. 2004; Klaunig and Kamendulis 2004).

In addition to free radical production, another source of indirect post-irradiation toxicity is inflammatory processes. Radiation-induced damage initiates pro-inflammatory reactions in the surrounding tissue resulting in the production of numerous pro-inflammatory cytokines and chemokines, like interleukin-1, interleukin-6, tumour necrosis factor α and transforming growth factor β (Kim et al. 2014). The pro-inflammatory response is primarily responsible for the long-term toxicity associated with radiation toxicity, whereas the free radical production is associated with the short-term toxicities (Graves et al. 2010; Kim et al. 2014).

Antioxidants can minimize the damaging effects of ROS by converting strong oxidants to less reactive forms (Sies 1997). Most research on antioxidants has focused on carotenoids (Sies et al. 1992). The most potent antioxidant among carotenoids seems to be lycopene (LYC), an acyclic isomer of beta-carotene (Di Mascio et al. 1989). It is synthesized by microorganisms, red fruits and vegetables, including tomatoes, watermelons, pink grapefruits, apricots, pink guavas and papaya (Stahl and Sies 1996). Tomato products (about 60–50%) and fresh tomatoes are the main sources of LYC in food rations (about 30–40%) (Wawrzyniak and Sitek 2010a, b). Humans absorb a significant portion of intact LYC directly, and it circulates through and accumulates in their plasma, liver and peripheral tissues (Wan 2012). The blood concentration of LYC (~1 μM) is highest among inhabitants of Italy and Greece (Al-Delaimyl et al. 2004). The concentration of LYC in tissues ranges from 0.2 to 21.4 nM/g tissue and mainly depends on tissue type, diet, bioavailability, effectiveness of lycopene excretion and activities of various lipoprotein receptors on the surface of the cells (Goralczyk and Siler 2004).

LYC has strong free radical scavenging properties and helps keeping the balance of endogenous defense systems of cells (Stahl and Sies 2003; Yapaing et al. 2002), so it is particularly promising as radiation modifier/protector. Such an agent could alter the response of tissues to radiation when it is present prior to or shortly after radiation exposure. In contrast to radioprotectors, radiomitigators are agents which have the capacity to minimize toxicity when applied after a radiation exposure (Cirin et al. 2010). The known radioprotectors cysteine and cysteamine are toxic at doses required for radioprotection (Velioglu-Ogunc et al. 2009). LYC is expected to be non-toxic and both act as radioprotector and radiomitigator.

During the past decades, numerous animal or in vitro studies have suggested that LYC reduces the risk of cancers of various organs (Giovannucci 1999; Gloria et al. 2014; Levy et al. 1995), retards the growth of tumours (Kobayashi et al. 1996; Nagasawa et al. 1995) and has health-promoting properties against other diseases, like osteoporosis (Rao et al. 2003), male infertility (Wertz et al. 2004), or cardiovascular diseases (Englehard et al. 2006; Huang et al. 2013; Li and Xu 2013; Rao and Agarwal 2000). Also, it has been shown that LYC may provide protection against mutations induced by ionizing radiation (Cavusoglu and Yalcin 2009). Lycopene has also been shown to have anti-inflammatory effects. In particular, it has been associated with downregulation of TNF-α gene expression and inhibition of TNF-α secretion (Bonvissuto et al. 2011; Marcotorchino et al. 2012).

In the past, lycopene was administered mainly before or simultaneously with irradiation (Andic et al. 2009; Saada and Azab 2001; Saada et al. 2010; Srinivasan et al. 2007, 2009). There are very few studies where animals or cells were treated by LYC after radiation (Forssberg et al. 1959; Meydan et al. 2011) and the aim of our study was to close this gap. We chose human peripheral lymphocytes as the mode system because lymphocytes are good markers of the actual body state and may be a reliable model for studying the effect of additions of specific antioxidants to the diet (Duthie et al. 1996; Zhang et al. 1991). We analysed the level of DNA damage because it is a useful biomarker of the oxidative status and the antioxidant defense system (Duthie et al. 1996). We examined the radioprotective and radiomitigating properties of lycopene which was administered before and after exposure to irradiation. Specifically, we investigated whether LYC, applied at appropriate intervals before and after exposure to ionizing radiation, can prevent radiation-induced DNA mutations and modulate DNA repair.

## Materials and methods

### Isolation of lymphocytes

Samples of human peripheral blood were aseptically collected in heparinized sterile tubes from a nonsmoking, healthy individual (female, 30 years) according to the procedure of Anderson et al. (1997).

For isolating the lymphocytes, whole blood was mixed 1:1 with phosphate-buffered saline (PBS). 5 ml of this mixture was cautiously placed on top of 2.5 ml of lympho separation medium (MP Biomedicals) and centrifuged at 918×*g* for 20 min at room temperature. The lymphocyte layer was removed, mixed with 10 ml PBS and centrifuged at 450×*g* rev/min for 10 min. Then, the supernatant was removed and the remaining cell pellet was shaken up and transferred to eppendorf tubes (50 µl of cell suspension to each tube).

### Preparation of lycopene

The stock solution of LYC was prepared as follows: 1 mg of LYC (purity >90%, ROTH GmbH, Germany, cat. no: 1180.1) was dissolved in 1 µl of dimethyl sulfoxide (DMSO). Dimethyl sulfoxide permeabilizes cell membranes and is traditionally used as a chemical penetration enhancer to deliver active molecules into cells (de Menorval et al. 2012). From the stock solution, the three different concentrations, namely 10, 20 and 40 µM/ml of LYC were added to lymphocytes in eppendorf tubes. The choice of doses for this study was based on previous studies where the effective concentration of LYC was determined at the level of at least 10 µM/ml (Saada et al. 2010; Srinivasan et al. 2007, 2009). The cell suspension was supplemented by PBS to 1 ml of solution in each eppendorf tube. The PBS buffer keeps the pH constant and its concentration of ions and osmotic pressure is comparable to that of human body fluids. Due to its isotonic nature and lack of live cell toxicity, it is widely used in many analyses (Scorpio 2000). The maximal concentration of DMSO in the tube was 2% and this dose was used as sham control. The controls for each dose of LYC and for the determination of viability assay of lymphocytes with trypan blue were prepared in a similar way.

### Treatment of the cells

The isolated lymphocytes (after determination of their viability) were exposed to X-radiation at doses of 0.5, 1 and 2 Gy. Control cells were unexposed. A therapeutic Roentgen unit Medicor type THX-250 was used as the X-ray source. It was operated with the following parameters: 155 kV, 18 mA, added filtration 0.25 mm Cu and HVL 2 mm Al. Lymphocytes were irradiated at the dose rate of 0.2 Gy/min. LYC, dissolved in DMSO at various doses, was added to test samples at different intervals before or after the irradiation (1 h before, immediately before, immediately after and 1 h after). The time intervals were chosen on the basis of references and our own unpublished preliminary study. We have used a combination of each X-ray dose (0.5, 1 and 2 Gy) with each LYC dose (10 µM/ml, 20 µM/ml, 40 µM/ml). Then the cells were incubated for 1 h in a water bath at 37 °C. At the same time control cells (negative controls), cells exposed to LYC only and to X-rays only were treated accordingly.

Three independent (*n* = 3) experiments were performed. The blood from the donor was taken at three different days within a period of 1 month. The level of DNA damage was evaluated using the alkaline comet assay.

### Comet assay

The impact of LYC on X-radiation-induced DNA damage in lymphocytes was studied using single cell gel electrophoresis (comet assay) according to the procedure of Singh et al. (1988) and Anderson et al. (1997). Each cell sample was centrifuged at 1778×*g* for 3 min and the supernatant was removed. 75 µl of 0.5% low-melting-point agarose (LMPA) at 37 °C was added to the pellet remaining in the Eppendorf tube, mixed and embedded onto glass microscope slides, which were previously covered with 1% normal-melting-point agarose (NMPA). Slides were covered with cover slips and put in a refrigerator (4 °C) to solidify the agarose. After solidification the cover slips were removed and another layer of LMPA was added. Slides were covered with cover slips and allowed to solidify at 4 °C again. Then, the cover slips were removed and slides were immersed in a lysing solution (2.5 M sodium chloride—NaCl, 100 mM ethylenediaminetetraacetic acid—EDTA, 10 mM Tris, 1% sodium lauryl sarcosinate, pH 10, plus 1% Triton-X and 10% dimethyl sulfoxide—DMSO) overnight at 4 °C. After that the slides were incubated in the electrophoresis solution (10 N NaOH, 200 mM EDTA—pH 10 in distilled water at 4 °C) for 20 min to allow DNA unwinding. Alkaline electrophoresis was conducted for 20 min at 4 °C, 0.6 V/cm and 300 mA. The level of the electrophoresis buffer was approximately 0.25 cm above the slides. After neutralization, the slides were stained with ethidium bromide (EtBr) and examined using fluorescence microscope. Images of 100 randomly selected lymphocytes from each sample were recorded and analysed using the CASP image analysis software (Końca et al. 2003). The DNA Tail Moment and Percentage of DNA in Comet Tail (% Tail DNA) were chosen as the parameters for analysis.

### Statistical analysis

One-way analysis of variance (ANOVA) was used to determine any significant differences between the results from various groups. Fisher’s post hoc test was applied to determine significant changes between groups. In both analyses, *p* values of <0.05 were considered significant.

## Results

All results are shown as mean values and standard deviations (SD) from three independent experiments. Although the level of DNA damage in DMSO-treated cells was somewhat higher than in the negative controls, the results were not statistically significant (*p* > 0.05). A dose-dependent increase of DNA damage was evident in lymphocytes exposed to X-rays alone.

**Fig. 1:**
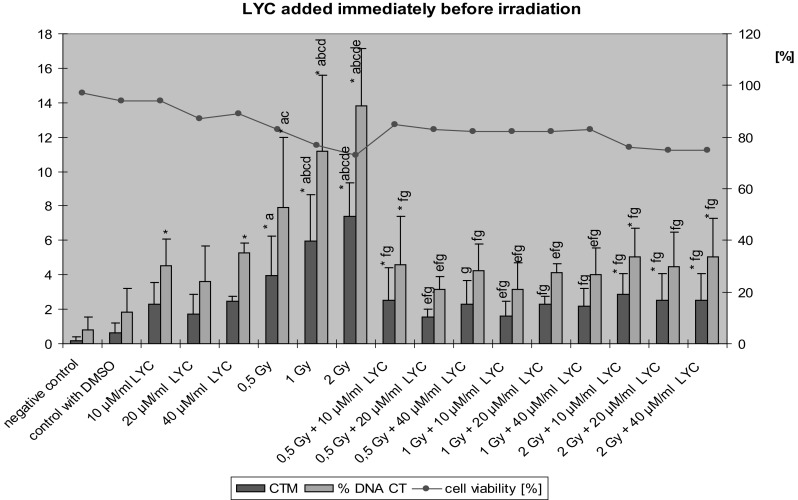
The value of Tail Moment (CTM) and Tail DNA  % (% DNA CT) in lymphocytes treatment by LYC 1 h before the exposure to ionizing radiation (*n* = 3); *p < 0.05 compared to control; ^a^ *p* < 0.05 compared to DMSO; ^b^ *p* < 0.05 compared to 10 µM LYC alone; ^c^ *p* < 0.05 compared to 20 µM LYC alone; ^d^ *p* < 0.05 compared to 40 µM LYC alone; ^e^ *p* < 0.05 compared to 0.5 Gy alone; ^f^ *p* < 0.05 compared to 1 Gy alone; ^g^ *p* < 0.05 compared to 2 Gy alone by post hoc Fisher’s test

After administration of LYC 1 h before exposure of lymphocytes to ionizing radiation, the values of Tail Moments and % Tail DNA were markedly lower than those in cells where LYC was added directly before the exposure to X-rays (Fig. 1). A significant decrease in the degree of DNA damage was observed especially at doses of 1 and 2 Gy combined with all doses of LYC. Only at the dose of 0.5 Gy + 40 µM/ml LYC, a statistically significant effect was not observed. However, exposure to LYC alone at a dose of 40 µM/ml caused a statistically significant increase of the Tail Moment and % Tail DNA as compared to negative controls. Similarly as in lymphocytes treated with LYC immediately before exposure to ionizing radiation, there were no significant differences in cells exposed to a combination of radiation and LYC at different doses.

**Fig. 2:**
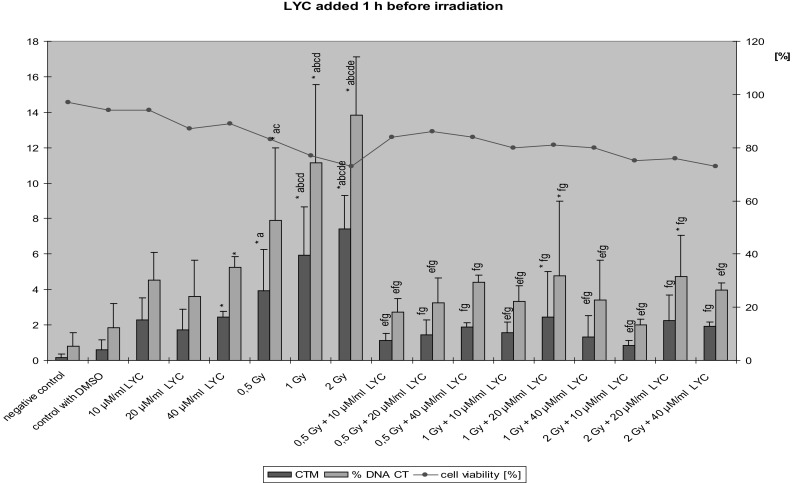
The value of Tail Moment (CTM) and Tail DNA % (% DNA CT) in lymphocytes treatment by LYC immediately before the exposure to ionizing radiation (*n* = 3); *p < 0.05 compared to control; ^a^ *p* < 0.05 compared to DMSO; ^b^ *p* < 0.05 compared to 10 µM LYC alone; ^c^ *p* < 0.05 compared to 20 µM LYC alone; ^d^ *p* < 0.05 compared to 40 µM LYC alone; ^e^ *p* < 0.05 compared to 0.5 Gy alone; ^f^ *p* < 0.05 compared to 1 Gy alone; ^g^ *p* < 0.05 compared to 2 Gy alone by post hoc Fisher’s test

Results obtained in lymphocytes treated with LYC immediately before exposure to ionizing radiation are shown in Fig. 2. Cell viability in all groups was higher than 70%. Radiation at all doses caused a statistically significant increase of DNA damage in lymphocytes as compared to negative controls and to cells treated with DMSO only. At doses of 0.5 Gy + 10 µM/ml LYC and 2 Gy with all doses of LYC, higher values of Tail Moment were also noted as compared to negative controls, but not to DMSO only treated cells. In the case of % Tail DNA the results were similar, except in the group of 2 Gy + 20 µM/ml LYC, where the increase of DNA damage was not statistically significant.

The results showed a significant decrease in the values of the Tail Moment and % Tail DNA in cells treated with various concentrations of LYC directly before exposure to X-rays as compared to groups exposed to irradiation alone. Especially at doses of 1 and 2 Gy, DNA damage was significantly reduced by all doses of LYC. After 0.5 Gy of ionizing radiation, a statistically significant decrease in the values of the Tail Moment and % Tail DNA was observed only with a dose of 20 µM/ml LYC. In contrast, in lymphocytes incubated only with LYC at doses of 10 and 40 µM/ml, a statistically significant increase of the values of % Tail DNA, but not of the Tail Moment, was noted in comparison to negative controls. There were no significant differences in cells exposed to a combination of radiation and LYC at different doses.

**Fig. 3:**
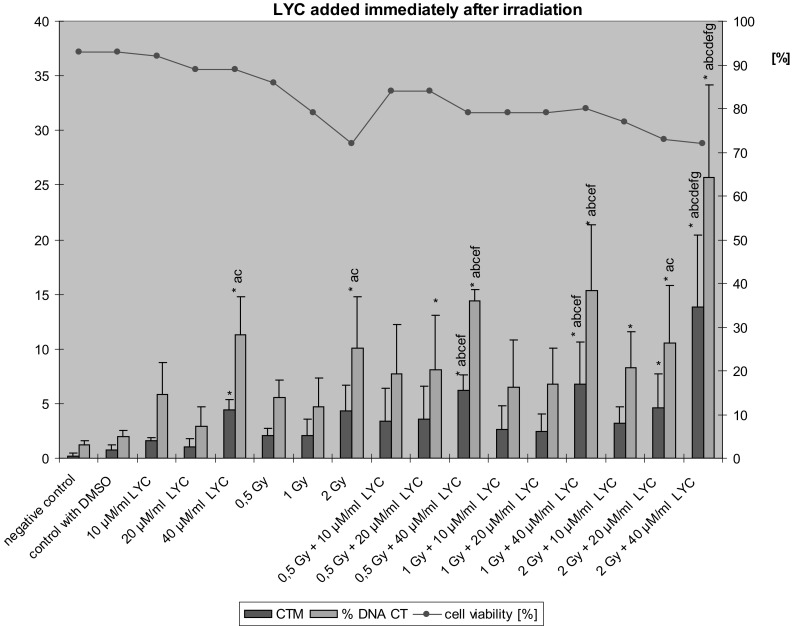
The value of Tail Moment (CTM) and Tail DNA  % (% DNA CT) in lymphocytes treatment by LYC immediately after the exposure to ionizing radiation (*n* = 3); *p < 0.05 compared to control; ^a^ *p* < 0.05 compared to DMSO; ^b^ *p* < 0.05 compared to 10 µM LYC alone; ^c^ *p* < 0.05 compared to 20 µM LYC alone; ^d^ *p* < 0.05 compared to 40 µM LYC alone; ^e^ *p* < 0.05 compared to 0.5 Gy alone; ^f^ *p* < 0.05 compared to 1 Gy alone; ^g^ *p* < 0.05 compared to 2 Gy alone by post hoc Fisher’s test

The Tail Moment and % Tail DNA values in lymphocytes treated with LYC immediately after exposure to ionizing radiation are shown in Fig. 3. When LYC was administered immediately after exposure to ionizing radiation, no decrease of DNA damage was noted. On the contrary, a dose of 40 µM/ml LYC significantly increased the effect of ionizing radiation. A similar effect was noted in cells treated only with LYC at dose of 40 µM/ml. Moreover, LYC at a dose of 40 µM/ml increased the level of  % Tail DNA in comparison to cells treated with DMSO alone or to a LYC dose of 20 µM/ml. Also, after a combined exposure to 2 Gy + 20 µM/ml LYC, an augmented value of Tail Moment was noted. 2 Gy + 10 µM/ml LYC and 2 Gy + 20 µM/ml LYC induced increased values of % Tail DNA as compared to negative controls.

**Fig. 4:**
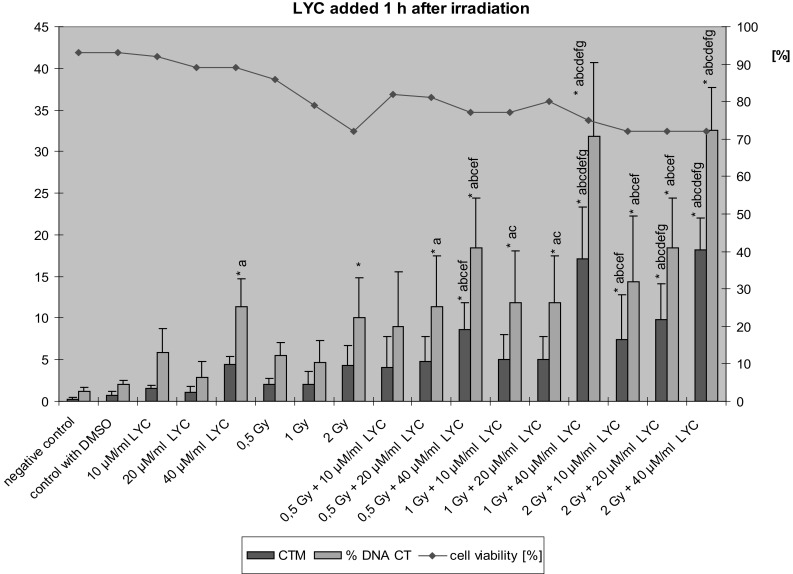
The value of Tail Moment (CTM) and Tail DNA  % (% DNA CT) in lymphocytes treatment by LYC 1 h after the exposure to ionizing radiation (*n* = 3); *p < 0.05 compared to control; ^a^ *p* < 0.05 compared to DMSO; ^b^ *p* < 0.05 compared to 10 µM LYC alone; ^c^ *p* < 0.05 compared to 20 µM LYC alone; ^d^ *p* < 0.05 compared to 40 µM LYC alone; ^e^ *p* < 0.05 compared to 0.5 Gy alone; ^f^ *p* < 0.05 compared to 1 Gy alone; ^g^ *p* < 0.05 compared to 2 Gy alone by post hoc Fisher’s test

The Tail Moment and % Tail DNA values in lymphocytes treated by LYC 1 h after exposure to ionizing radiation are shown in Fig. 4. Also in this experiment, the dose of 40 µM/ml LYC caused a significant increase of the Tail Moment. Administration of LYC 1 h after exposure to X-rays did not protect the lymphocytes against radiation-induced damage. On the contrary, a significant increase of the values of Tail Moment and % Tail DNA as compared to the control groups and to lymphocytes exposed to radiation alone was noted. Especially the dose of 40 µM/ml LYC, added after irradiation, resulted in a high level of DNA damage.

## Discussion

It was shown that ionizing radiation induces oxidative stress through the generation of ROS leading to an imbalance of pro- and antioxidants in exposed cells (Ateşşahin et al. 2006). Overproduction of ROS can lead to chromosomal damage and gene mutations (Cooke et al. 2003). Carotenoids have been shown to decrease the oxidative stress caused by aerobic metabolism (Bendich and Olson 1989; Britton 1995). A study by Di Mascio et al. (1989) confirmed that LYC is the antioxidant among carotenoids with the highest ability to quench singlet oxygen and trap peroxyl radicals. During past decades, numerous animal and in vitro studies have also suggested that LYC may provide protection against mutations induced by ionizing radiation. LYC has been mainly administered before or simultaneously with radiation exposure. A few studies described treatment of cells (Cavusoglu and Yalcin 2009; Srinivasan et al. 2007, 2009) or animals (Andic et al. 2009; Forssberg et al. 1959; Saada and Azab 2001; Saada et al. 2010) by LYC after irradiation. The aim of our study was to investigate the protective action of LYC administered before and after exposure to radiation.

Somewhat surprisingly, a statistically significant increase of DNA damage was noted in cells treated by LYC alone at a dose of 40 µM/ml, suggesting that at this concentration LYC is genotoxic. Lowe et al. (1999) found that LYC concentration of >3 µM/L acts as a prooxidant in HT29 cells. Also, Yeh and Hu (2000) noted that there was a slight but not dose-dependent increase in comet formation induced by LYC without additional oxidant treatment. They suggested that this slight increase may be attributed to the presence of some pre-formed auto-oxidative products or the products formed during incubation. This observation was confirmed in a follow-up study where oxidized LYC leads to oxidative damage to both purified DNA and cellular DNA (Yeh and Hu 2001). It has also been demonstrated that at high concentrations, carotenoids exhibit a tendency to aggregate or crystallize out of solution, with different compounds behaving differently, depending on their structure (Gruszecki 1999; Ruban et al. 1993). The biophysical and chemical properties of aggregates are quite different from those of the monomeric form of the carotenoid in solution (Britton 1995; Gruszecki 1999).

LYC, added to cells immediately before radiation exposure, significantly reduced the level of DNA damage. However, the level of protection was even higher when LYC was added 1 h prior to irradiation. A significant decrease in the degree of DNA damage was observed especially after X-ray doses of 1 and 2 Gy in combination with all doses of LYC. These results fit well with earlier studies, where it was shown that pretreatment with LYC resulted in a decrease of radiation-induced lipid peroxidation and improved antioxidant status, preventing induction of DNA damage (Srinivasan et al. 2009). It is also known that LYC reduces the toxicity of cisplatin which induces lipid peroxidation in rat testis (Atessahin et al. 2006). The possible mechanism by which carotenoids can quench singlet oxygen is via energy transfer from singlet oxygen to the LYC molecule, converting it to an energy-rich triplet state (Wertz et al. 2004). LYC, administered before X-irradiation, was shown to protect mice from lethal bacterial infections which killed irradiated and infected mice (Forssberg et al. 1959). Also, the protective effect of LYC on radiation-induced intestinal toxicity was investigated (Saada et al. 2010; Andic et al. 2009). Both studies showed that LYC acts protectively against intestinal toxicity by reducing lipid peroxidation and increasing antioxidant enzyme activity. Finally, another study showed that pretreatment of γ-irradiated lymphocytes with LYC resulted in decrease of lipid peroxidation and improved antioxidant status, preventing damage to lymphocytes (Srinivasan et al. 2007).

Irrespective of whether LYC was added 1 h or immediately before irradiation, we did not observe any relationship between its dose the protective effect on radiation-induced damage. As already mentioned, it may be caused with the tendency of LYC to aggregate or crystallize out of solution. Srinivasan et al. (2007) also noted that the higher dose was less effective because its concentration may have resulted in the production of byproducts, which may have interfered with the antioxidant activity of LYC, thus decreasing its effect. Our results may be such also by the difference in the source of LYC. Most of studies provided evidence for antioxidant properties of LYC have been performed with LYC extract (Kelkel et al. 2011). We used pure LYC to our study.

As already mentioned in the introduction, ionizing radiation induces long-lived radicals and inflammatory responses which are associated with chronic toxicity. LYC has both antioxidant and anti-inflammatory properties and there are very few studies where the effect of LYC was investigated when applied after irradiation (Forssberg et al. 1959; Meydan et al. 2011). In our study, the administration of LYC after exposure to X-rays caused a significant increase of DNA damage, especially at the dose of 40 µM/ml. As mentioned above, Lowe et al. (1999) showed that LYC lost the ability to protect cells against oxidative damage at higher concentrations. Also, Eichler et al. (2002) reported that LYC protects skin fibroblasts from UV-induced formation of TBARS (thiobarbituric acid-reactive substances) only at a concentration up to 0.15 nM/mg, whereas at higher concentrations, a pro-oxidant effect could be observed. Moreover, it has been suggested that carotenoids might exert pro-oxidant effects depending both on their concentration and the partial O_2_ pressure (pO_2_) (Young and Lowe 2001; Burton and Ingold 1984; Eichler et al. 2002). However, most of this data are related to β-carotene and it has been proposed that the pro-oxidant effects of carotenoids (or rather the lack of antioxidant effects) may be due to their autoxidation (Handelman et al. 1991; Liebler and Kennedy 1992; King et al. 1997; Baker et al. 1999; Kennedy and Liebler 1991). Burton and Ingold (1984) first demonstrated that the antioxidant behaviour of β-carotene was, in part, dependent upon the partial pressure of oxygen. They showed that at low pO_2_, β-carotene acted as a chain-breaking antioxidant (consuming peroxy radicals), while at higher pO_2_ the carotenoid lost its antioxidant ability and actually exhibited pro-oxidant behaviour due to autooxidation (the carotenoid radical could react with oxygen to produce a carotenoid peroxyl radical autoxidation, which is capable of acting as a pro-oxidant). Also, El-Agamey et al. (2004) showed that carotenoids could lose their antioxidant activity at high concentrations and/or at high pO_2_. Both the antioxidant and pro-oxidant effects of LYC were dependent also on the source carotenoid and oxidants used and also upon their interaction with other co-antioxidants, especially vitamins E and C (Gajowik and Dobrzyńska 2014; Kelkel et al. 2011; Yeh and Hu 2000; Young and Lowe 2001). However, this does not necessarily mean that unlike in vitro system, carotenoids act as pro-oxidants under in vivo conditions. Meydan et al. (2011) showed that LYC pretreatment significantly reduces the increase in lipid peroxidation and reduces the lowering of levels of GSH, and GSH-Px and SOD enzyme activities in liver 48 h after RT and also long term (up to 60 days after RT). These results showed that continued treatment with LYC might be useful to reduce oxidative damage caused by radiotherapy in rats. However, Jomova et al. (2012) point to the importance of mapping of experimental conditions under which carotenoids may behave as pro-oxidants.

## Conclusions

In summary, the present study in human lymphocytes confirms that pretreatment with LYC protects DNA against damage induced by ionizing radiation. All tested doses of LYC protected lymphocytes against the genotoxic effect of X-rays. However, when added after radiation exposure, LYC had no ability to reduce the level of DNA damage. On the contrary, especially at the dose of 40 µM/ml, LYC induced DNA damage demonstrating a pro-oxidant activity. Nevertheless, supplementation with LYC, especially at low doses, may be useful in protection from radiation-induced oxidative damage.
